# The Impact of Machine Learning and Robot-Assisted Gait Training on Spinal Cord Injury: A Systematic Review and Meta-Analysis

**DOI:** 10.3390/jcm12237230

**Published:** 2023-11-22

**Authors:** Dewa Putu Wisnu Wardhana, Sri Maliawan, Tjokorda Gde Bagus Mahadewa, Rohadi Muhammad Rosyidi, Sinta Wiranata

**Affiliations:** 1 Neurosurgery Division, Department of Surgery, Faculty of Medicine, Universitas Udayana, Prof. Dr. IGNG Ngoerah General Hospital, Denpasar 80113, Indonesia; 2 Department of Neurosurgery, Medical Faculty, Mataram University, West Nusa Tenggara General Hospital, Mataram 84371, Indonesia; 3 Faculty of Medicine, Universitas Udayana, Denpasar 80232, Indonesia

**Keywords:** spinal cord injury, machine learning, robot-assisted gait training

## Abstract

Introduction: Spinal cord injury (SCI) is a significant and transforming event, with an estimated annual incidence of 40 cases per million individuals in North America. Considering the significance of accurate diagnosis and effective therapy in managing SCI, Machine Learning (ML) and Robot-Assisted Gait Training (RAGT) technologies hold promise for enhancing optimal practices and elevating the quality of care. This study aims to determine the impact of the ML and RAGT techniques employed on the outcome results of SCI. Methods: We reviewed four databases, including PubMed, Scopus, ScienceDirect, and the Cochrane Central Register of Controlled Trials (CENTRAL), until 20 August 2023. The keywords used in this study encompassed the following: a comprehensive search was executed on research exclusively published in the English language: machine learning, robotics, and spinal cord injury. Results: A comprehensive search was conducted across four databases, identifying 2367 articles following rigorous data filtering. The results of the odd ratio (OR) and confidence interval (CI) of 95% for the ASIA Impairment Scale, or AIS grade A, were 0.093 (0.011–0.754, *p* = 0.026), for AIS grade B, 0.875 (0.395–1.939, *p* = 0.743), for AIS grade C, 3.626 (1.556–8.449, *p* = 0.003), and for AIS grade D, 8.496 (1.394–51.768, *p* = 0.020). The robotic group exhibited a notable reduction in AS (95% CI = −0.239 to −0.045, *p* = 0.004) and MAS (95% CI = −3.657 to −1.066, *p* ≤ 0.001) measures. This study also investigated spasticity and walking ability, which are significant. Conclusions: The ML approach exhibited enhanced precision in forecasting AIS result scores. Implementing RAGT has been shown to impact spasticity reduction and improve walking ability.

## 1. Introduction

Spinal cord injury (SCI) is a significant and transforming event, with an estimated annual incidence of 40 cases per million individuals in North America [1]. Following the occurrence of an injury, there are physiological repercussions that impact several body systems and are frequently accompanied by a substantial risk of mortality. Previous studies have reported inconsistent findings about the rates of in-hospital mortality, which have been shown to range from 3% to 13%. Similarly, the 1 year mortality rates after SCI have been estimated to range from 5% to 10% [2,3,4,5]. Numerous research studies have previously elucidated predictive features and algorithms for evaluating the probability of death after SCI. However, there is currently a shortage of prognostic instruments tailored specifically to the SCI patient cohort that may be conveniently employed in a clinical environment [2,5,6,7,8]. Apart from its utility in informing clinical decision-making and facilitating patient and family talks, a predictive tool may also serve as a valuable instrument in clinical research by accounting for the possible influence of distinct patient and injury variables on the mortality risk of study participants.

Considering the significance of accurate diagnosis and effective therapy in managing SCI, Machine Learning (ML) and Robot-Assisted Gait Training (RAGT) technologies hold promise for enhancing optimal practices and elevating the quality of care. The numerical value is provided by the user [9,10]. ML is often regarded as the most promising field within the domain of artificial intelligence (AI). It encompasses using algorithms to automatically generate predictions or outputs by analyzing the attributes of given inputs [11]. ML possesses inherent advantages in processing large datasets compared to traditional statistical approaches. They exhibit greater precision and reproducibility than conventional models and even skilled operators. ML can potentially uncover nuanced information that may not be perceptible to the human eye in specific image-related activities [11,12]. The user’s text is too short to be rewritten academically. In the current era characterized by large-scale datasets, ML techniques can significantly enhance diagnostic accuracy and prognosis [13].

Clinicians consistently face challenges in rehabilitating patients to improve pain management, reduce stiffness, and increase walking capacity. The application of RAGT in rehabilitation has experienced increased prevalence due to its ability to transcend the constraints imposed by the extent of an individual’s muscle paralysis. The provision of recurrent and functional task training by RAGT has been found to elicit increased activity in the sensorimotor cortex (specifically, S1 and S2) and the cerebellar areas [14,15]. The convergence of advancements in fundamental neuroscience and technology innovation has presented neurosurgery with distinctive prospects for utilizing ML and RAGT in research and clinical settings to enhance patient care [16].

Moreover, using customized or precision medicine in the context of patients with SCI seems beneficial in customizing expectations and treatment strategies, considering the intrinsic diversity observed within this group in terms of outcomes, functional prognosis, and the rehabilitation process [17,18,19]. This study comprehensively aims to examine the impact of ML on predicting AIS score outcomes and RAGT on rehabilitation outcomes. The focus was on research endeavors to enhance therapeutic advancements and develop predictive models.

## 2. Material and Methods

### 2.1. Search Strategy

The PRISMA [20] systematically reviewed four databases, including PubMed, Scopus, ScienceDirect, and the Cochrane Central Register of Controlled Trials (CENTRAL), until 20 August 2023. The MeSH phrases and keywords used in this study encompassed the following: a comprehensive search was executed on research exclusively published in the English language: machine learning, robotics, and spinal cord injury. The reference lists of the published works were examined to identify potential areas for further investigation. In instances where duplicate studies were identified, preference has been given to studies with larger sample sizes. Each study produced the subsequent findings: (1) the initial name and year of publication; (2) the nation and total sample; (3) type of study design; (4) level of injury; (5) ASIA Impairment Scale (AIS) grade; (6) intervention; and (7) outcome.

### 2.2. Data Selection

Three reviewers (D.P.W.W., S.M., and T.G.B.M.) independently performed the selection. The conflict among the first three reviewers was settled by the establishment of a consensus by the fourth and fifth reviewers. Exclusion of studies occurred in cases where essential outcome measures were absent or not assessed. The included papers should be: (1) a paper that investigated ML and RAGT; (2) research given information on ML and RAGT as well as outcome status; (3) studies that provide the computation data for the calculation of the total sample, mean, and standard deviation (SD); and (4) a full-text article. The protocol for this review was registered in PROSPERO, with the registration number CRD42023464103. The publication was subsequently developed according to PRISMA principles.

### 2.3. Data Extraction

The relevant data were extracted using a pre-established Google Sheets Excel Online form by two reviewers (D.P.W.W. and S.W.) who worked independently. Any discrepancies were identified and resolved through consensus with a senior reviewer (S.M.). When data was absent or doubts arose, we initiated electronic correspondence with the authors via email to acquire the necessary data.

### 2.4. Risk of Bias

Two authors (D.P.W.W. and S.M.) independently evaluated the bias quality of the chosen randomized controlled trials (RCTs) using the Cochrane risk of bias assessment methodology [20]. Two authors have used the Newcastle Ottawa Scale (NOS) [21] to evaluate the chosen articles’ methodological quality independently. We divided the articles’ overall quality into moderate (4–6) and high (7–9). Any potential conflicts were effectively resolved by open dialogue and the attainment of mutual agreement facilitated by the involvement of the third author (S.W.).

### 2.5. Statistical Analysis

RCTs and non-RCTs were categorized into separate groups and subjected to individual studies afterward. The treatment impact was analyzed using Comprehensive Meta-Analysis (CMA) version 3 through statistical analysis. The mean differences (MD), odds ratio (OR), and 95% CI were computed for outcome measures. We use a random effect model for the analysis. The application of a random effect model offers distinct advantages over a fixed effect model due to its ability to effectively capture the entirety of the population under study.

## 3. Results

### 3.1. Search Results and Study Characteristics

**Figure 1:**
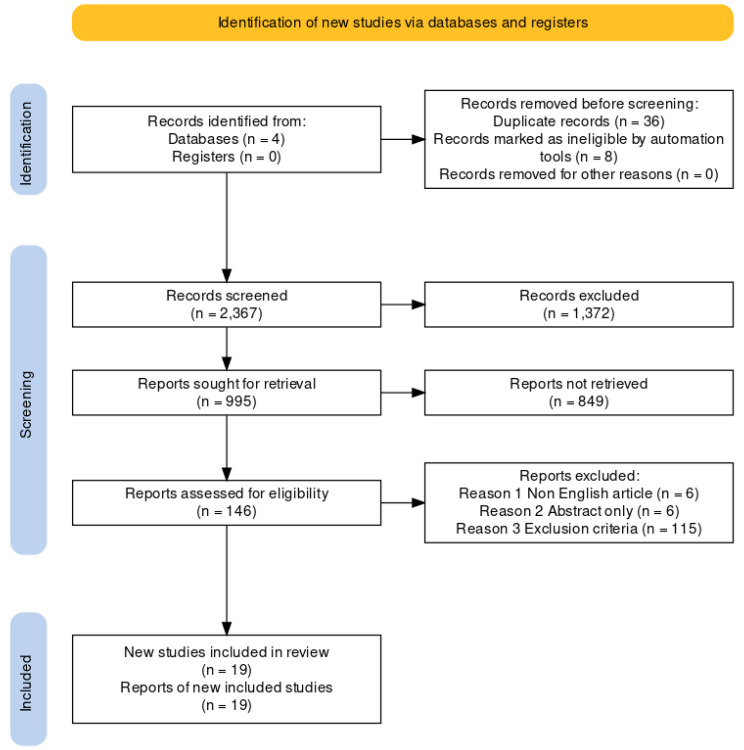
The flowchart depicts the process of selecting studies.

A comprehensive search was conducted across four databases, identifying 2367 articles following rigorous data filtering. A cumulative sum of 127 papers was deemed ineligible for inclusion in the study due to their failure to match the predetermined criteria for research inclusion in Figure 1. Ultimately, a total of 19 publications were selected for further research. The combined sample size of the papers included in this study was 1508 patients. The sample comprised 16 publications utilizing ML techniques and three articles using RAGT techniques. The articles were sourced from various nations, including the USA, Canada, Japan, Italy, Spain, Republic of Korea, and Switzerland.

The analysis incorporated both RCT and non-RCT study designs. The outcomes examined in the RAGT group encompassed measures such as the Ashworth Scale (AS), Modified Ashworth Scale (MAS), Visual Analog Scale (VAS), Lower Extremity Motor Score (LEMS), 6 Minute Walk Test (6MWT), 10 Meter Walk Test (10MWT), and Timed Up and Go Test (TUG). In the context of the ML group, an examination was conducted on the AIS grade result. The highest marks obtained from the collection of 13 articles are AIS C and D. The OR (95% CI) analysis was employed for the ML group. In contrast, the mean differences (MD) were utilized for the RAGT group. The features of the studies are presented in Table 1 and Table 2.

### 3.2. Bias Assessment

**Figure 2:**
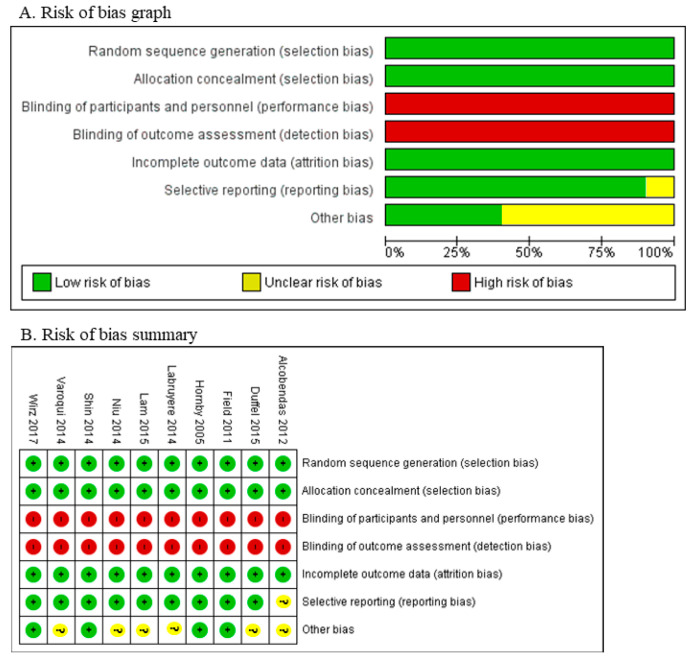
The risk of bias include the study are Alcobendas et al., 2012 [25]; Duffel et al., 2015 [32]; Field et al., 2011 [24]; Hornby et al., 2005 [22]; Labruyère et al., 2014 [28]; Lam et al., 2015 [33]; Niu et al., 2014 [29]; Shin et al., 2014 [30]; Varoqui et al., 2014 [31]; Wirz et al., 2017 [37].

All studies considered in the analysis demonstrate a minimal likelihood of selection bias. The existence of a wide range of rehabilitation procedures in numerous research studies has led to a significant occurrence of performance and detection bias. All research investigations demonstrate a low-risk level of attrition and reporting bias. Several studies exhibit a lack of clarity concerning potential biases, including issues related to loss of follow-up in Figure 2, Table 1 and Table 2.

### 3.3. Analysis of the ML Group

The analysis of all AIS grades A, B, C, and D included three RCT articles that fulfilled the inclusion criteria. We gathered data on the ability of ML to forecast outcomes based on the AIS score upon the patient’s initial hospital admission. We categorized these results into two groups: unimproved and improved in the AIS score. According to the findings of a meta-analysis, predicting using ML in SCI patients with AIS grade A does not improve their condition after re-evaluation following therapy. It may happen due to the presence of a complete injury. The results of the OR CI 95% for AIS grade A were 0.093 (0.011–0.754, *p* = 0.026).

**Figure 3:**
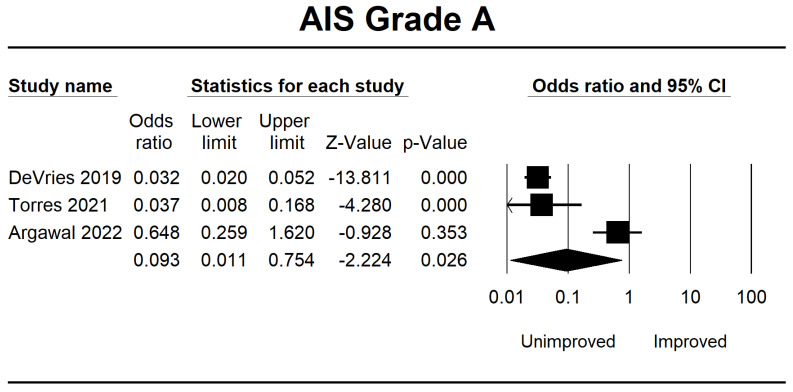
Forest plot of AIS grade A using OR ratio analysis between unimproved and improved prediction groups. The square box represents the point estimate for the respective study, while the horizontal line is the 95% CI. The diamonds represent pooled results. DeVries et al., 2009 [38]; Torres et al., 2021 [39]; Agarwal et al., 2022 [40].

**Figure 4:**
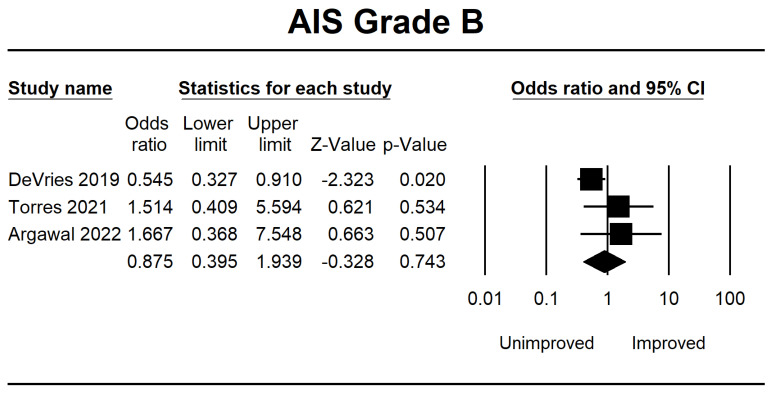
Forest plot of AIS grade B using OR ratio analysis between unimproved and improved prediction groups. The square box represents the point estimate for the respective study, while the horizontal line is the 95% CI. The diamonds represent pooled results. DeVries et al., 2009 [38]; Torres et al., 2021 [39]; Agarwal et al., 2022 [40].

**Figure 5:**
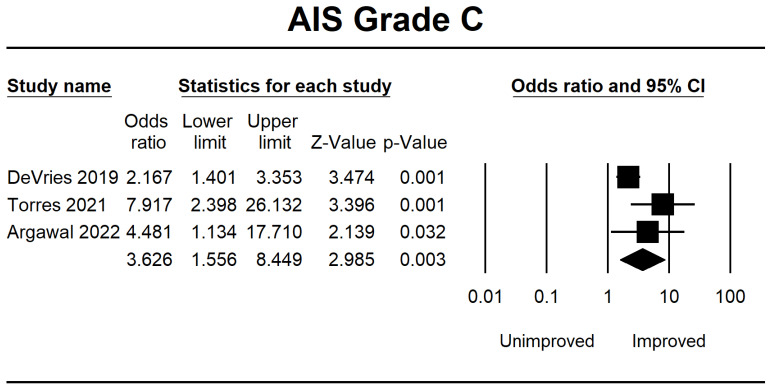
Forest plot of AIS grade C using OR ratio analysis between unimproved and improved prediction groups. The square box represents the point estimate for the respective study, while the horizontal line is the 95% CI. The diamonds represent pooled results. DeVries et al., 2009 [38]; Torres et al., 2021 [39]; Agarwal et al., 2022 [40].

**Figure 6:**
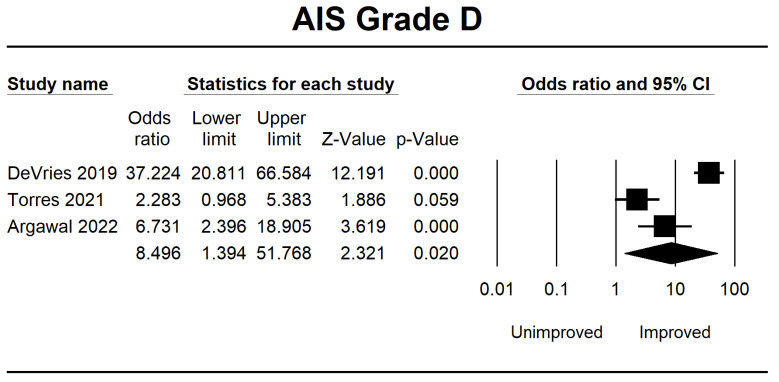
Forest plot of AIS grade D using OR ratio analysis between unimproved and improved prediction groups. The square box represents the point estimate for the respective study, while the horizontal line is the 95% CI. The diamonds represent pooled results. DeVries et al., 2009 [38]; Torres et al., 2021 [39]; Agarwal et al., 2022 [40].

Meanwhile, in AIS B, several patients demonstrated progress in the forest plot, but this outcome is because there is no significant difference of 0.875 (0.395–1.939, *p* = 0.743). Considerable improvement in AIS grade C was 3.626 (1.556–8.449, *p* = 0.003), and AIS grade D was 8.496 (1.394–51.768, *p* = 0.020). The final result is shown in Figure 3, Figure 4, Figure 5 and Figure 6.

### 3.4. Analysis of the RAGT Group

**Figure 7:**
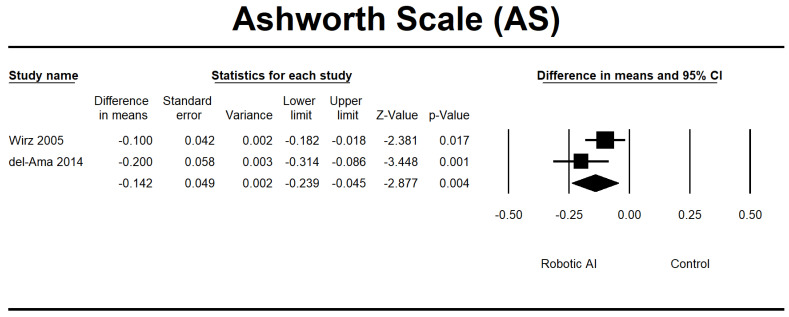
Forest plot of AS using standardized mean difference analysis between robotic and control groups. The square box represents the mean differences for the respective study, while the horizontal line is the 95% CI. The diamonds represent pooled results. Wirz et al., 2005 [23]; del-Ama et al., 2014 [27].

**Figure 8:**
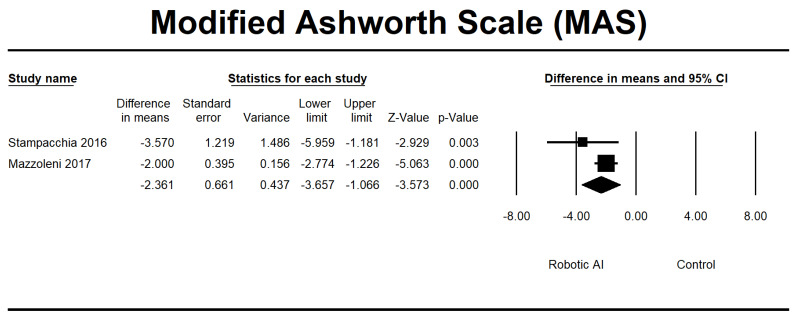
Forest plot of MAS using standardized mean difference analysis between robotic and control groups. The square box represents the mean differences for the respective study, while the horizontal line is the 95% CI. The diamonds represent pooled results. Stampacchia et al., 2016 [34]; Mazzoleni et al., 2017 [35].

Four RCTs [23,28,30,32] were conducted to evaluate the effects of interventions on spasticity. In the conducted investigations, all participants’ spasticity levels were categorized as mild, as indicated by a MAS score ranging from 0 to 2. Furthermore, it was noted that there were no significant alterations in spasticity levels following the implementation of RAGT. The robotic group exhibited a notable reduction in AS (95% CI = −0.239 to −0.045, *p* = 0.004) and MAS (95% CI = −3.657 to −1.066, *p* ≤ 0.001) measures. The pooled MD using MAS and AS was −2.149 and −0.142, respectively (Figure 7 and Figure 8).

**Figure 9:**
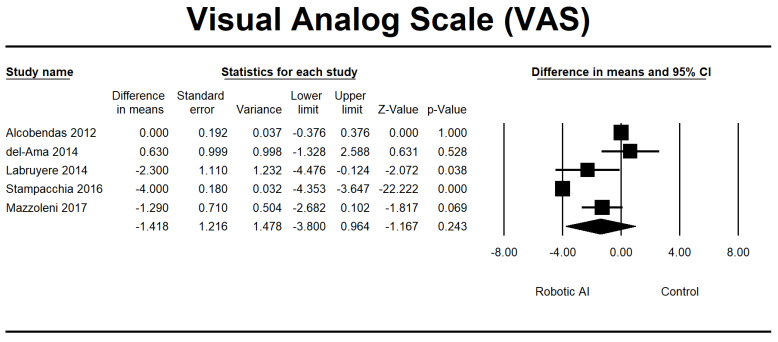
Forest plot of VAS using standardized mean difference analysis between robotic and control groups. The square box represents the mean differences for the respective study, while the horizontal line is the 95% CI. The diamonds represent pooled results. Alcobendas et al., 2012 [25]; del-Ama et al., 2014 [27]; Labruyère et al., 2014 [28]; Stampacchia et al., 2016 [34]; Mazzoleni et al., 2017 [35].

We also analyzed the pain parameter using the VAS variable. The findings from the analysis of the primary outcomes of pain following RAGT are depicted in Figure 9, consisting of two RCTs [28,32] and three non-RCTs [22,29,37]. Despite the observed trend indicating a potential reduction in pain in the robotic group, there was no statistically significant difference between the robotic and control groups. This lack of significance was consistent in the analysis (*p* = 0.243). The pooled MD was −1.418. The studies included in the research reported various pain levels, varying from mild to moderate.

**Figure 10:**
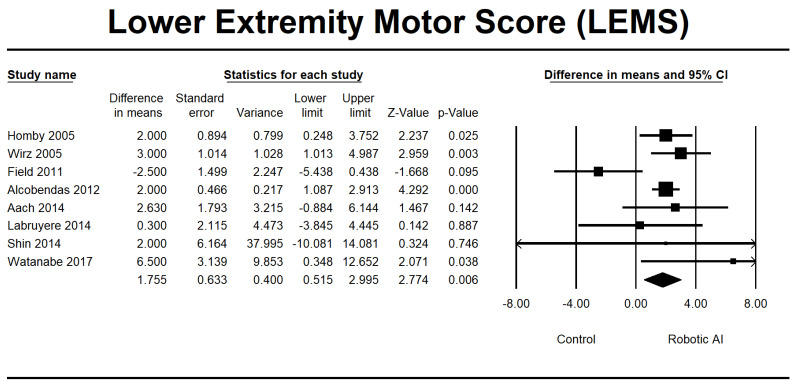
Forest plot of LEMS using standardized mean difference analysis between robotic and control groups. The square box represents the mean differences for the respective study, while the horizontal line is the 95% CI. The diamonds represent pooled results. Hornby et al., 2005 [22]; Wirz et al., 2005 [23]; Field et al., 2011 [24]; Alcobendas et al., 2012 [25]; Aach et al., 2014 [26]; Labruyère et al., 2014 [28]; Shin et al., 2014 [30]; Watanabe et al., 2019 [36].

**Figure 11:**
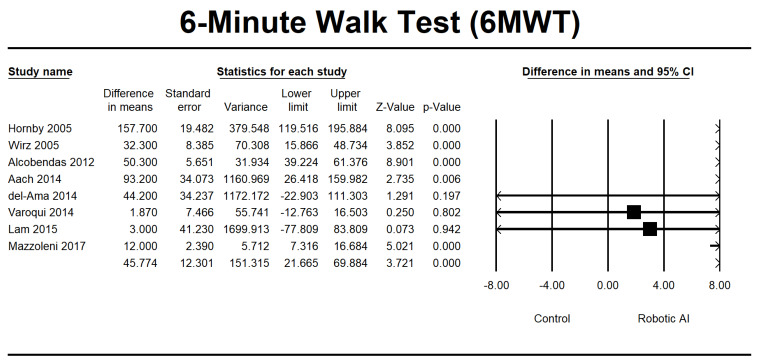
Forest plot of 6MWT using standardized mean difference analysis between robotic and control groups. The square box represents the mean differences for the respective study, while the horizontal line is the 95% CI. The diamonds represent pooled results. Hornby et al., 2005 [22]; Wirz et al., 2005 [23]; Alcobendas et al., 2012 [25]; Aach et al., 2014 [26]; del-Ama et al., 2014 [27]; Varoqui et al., 2014 [31]; Lam et al., 2015 [33]; Mazzoleni et al., 2017 [35].

**Figure 12:**
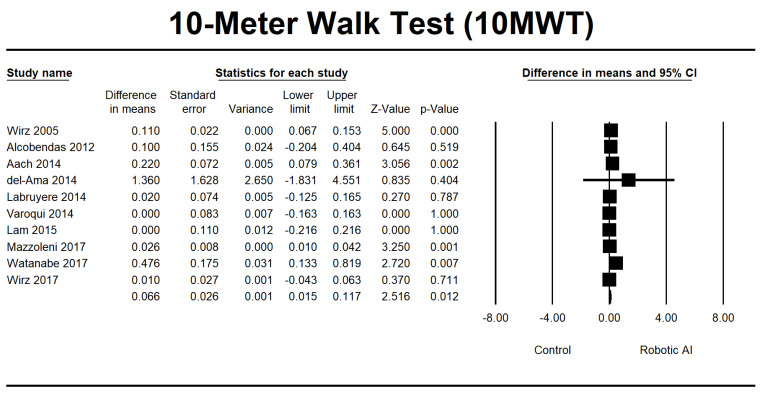
Forest plot of 10MWT using standardized mean difference analysis between robotic and control groups. The square box represents the mean differences for the respective study, while the horizontal line is the 95% CI. The diamonds represent pooled results. Wirz et al., 2005 [23]; Alcobendas et al., 2012 [25]; Aach et al., 2014 [26]; del-Ama et al., 2014 [27]; Labruyère et al., 2014 [28]; Varoqui et al., 2014 [31]; Lam et al., 2015 [33]; Mazzoleni et al., 2017 [35]; Watanabe et al., 2019 [36]; Wirz et al., 2017 [37].

**Figure 13:**
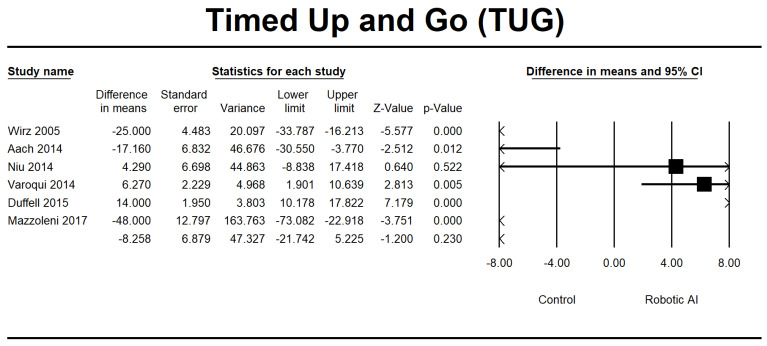
Forest plot of TUG using standardized mean difference analysis between robotic and control groups. The square box represents the mean differences for the respective study, while the horizontal line is the 95% CI. The diamonds represent pooled results. Wirz et al., 2005 [23]; Aach et al., 2014 [26]; Niu et al., 2014 [29]; Varoqui et al., 2014 [31]; Duffel et al., 2015 [32]; Mazzoleni et al., 2017 [35].

This study investigated walking ability by combining the LEMS, 6MWT, 10MWT, and TUG group analyses. In the LEMS analysis, we found five RCTs [22,24,25,28,29] and three non-RCTs [23,26,37] with statistically significant beneficial effects in favor of the robotic group (95% [CI] = 0.515 to 2.995, *p* ≤ 0.05). The mean difference is 1.755, as depicted in Figure 10. The 6MWT is a commonly used assessment tool in four RCTs [22,25,30,32], and four non-RCTs [23,26,27,34] were conducted to evaluate the 6MWT. Irrespective of the type of study design, there was a significant increase in walking distance in the group that received robotic assistance. The CI is 95% (21.665–69.884, *p* ≤ 0.001), with MD 45.774, as shown in Figure 11. The 10MWT comprises five RCTs [25,28,30,32,36] and five non-RCTs [23,26,27,34,37]. The 10MWT demonstrated a substantial improvement in the robotic group, as indicated by CI 95% 0.015–0.117, *p* = 0.012, with MD 0.066, as shown in Figure 12. The TUG study comprised a total of three RCTs [28,30,31]. The findings indicated a noteworthy enhancement in favor of the robotic group, with a CI of 95% −21.742 to 5.225, *p* = 0.230. The MD obtained by pooling the data using a random effects model was −8.258, as shown in Figure 13.

## 4. Discussion

The findings demonstrated encouraging outcomes in forecasting the improvement of AIS. Clinicians have used the AIS to categorize SCI and assess the extent of recovery. It may involve documenting enhancements, such as an improvement in AIS grade or deteriorations [41]. Within the confines of a conventional clinical environment, the primary determinants recognized for the prognostication of SCI recovery encompass patient age, patient gender, duration of hospitalization, manner of hospital release, SCI classification, procedural timing, nature of procedure, and presence of comorbidities. The prognosis of SCI is typically assessed using bedside evaluation and MRI or through classic clinical analysis, such as the calculation of odds ratios [42]. Hence, it is possible to identify the factors that can predict the recovery of SCI by incorporating the initial AIS scores into ML algorithms. This framework leverages big data and precision medicine, serving as a valuable tool for clinicians to enhance the overall prognosis of SCI patients. The current ML study [43] demonstrates a higher test accuracy of 73.6% than the MRI accuracy of 71.4%. A few studies [43,44,45,46,47] collectively utilize a patient sample size that is one to two times larger and incorporates a comprehensive evaluation of feature importance. Furthermore, even considering all AIS grades and employing a far less complex model that can be readily implemented, the outcomes are generally similar or superior.

The reduction of spasticity can be attributed to various theoretical frameworks. The RAGT technique elicits rhythmic movements in the lower limbs and offers sensory input. Prior research has indicated that rhythmic passive exercise has the potential to cause the reorganization of spinal circuitry and reduce stiffness in individuals with SCI [12]. The potential impact of repetitive elements within a therapy program on the enhancement of spasticity and locomotor function through the stimulation of spinal locomotor centers has been suggested [48]. Repetitive functional task training, sometimes called RAGT, represents a form of intervention that involves repeating available tasks. The mechanisms above could explain the observed reduction in spasticity resulting from RAGT [48]. As previously mentioned, despite decreasing spasticity, RAGT improves the detection of rhythmic muscle activations.

Furthermore, it is worth considering the significance of weight bearing as a contributing component. RAGT offers assistance that enables individuals to apply load to their lower extremities while engaging in training activities. The application of weight bearing on the lower limbs and the subsequent increase in muscle activation can positively impact the recovery of lower extremity motor function in individuals with LEMS. Furthermore, the findings of this meta-analysis indicate that the 6MWT can enhance endurance levels without imposing the strain associated with deliberate muscular contractions [34]. As evidenced by the findings of the LEMS improvement results, enhanced lower extremity strength probably contributes to an augmentation in walking speed, as observed in the 10MWT variable [49].

## 5. Limitations

One constraint of the analysis is the methodology employed for data collection in non-RCTs, particularly in the context of informing predictive modeling. It is generally acknowledged that prospective approaches are more suitable for developing accurate predictive models. The existing body of literature on this subject is minimal and exhibits variability in both topic and research design, hindering the possibility of conducting a meta-analysis or facilitating direct comparisons. Undoubtedly, the degree of injury has been demonstrated as a fundamental determinant in predicting the long-term functional result. Ultimately, models must undergo external validation and be meticulously implemented before their utilization and dependence in clinical settings. Regrettably, the identified articles lacked specific descriptions of the symptoms exhibited by patients. Furthermore, most of the research evaluated treatment efficacy solely during a predetermined timeframe, impeding our ability to examine this aspect comprehensively. The limitations of our study include the potential for future research to explore and offer additional insights into the symptoms and follow-up duration.

## 6. Conclusions

The ML approach exhibited enhanced precision in forecasting AIS result scores. The implementation of RAGT has been shown to positively impact the reduction of spasticity and the improvement of walking ability. The implementation of RAGT has been proven to be beneficial in the normalization of muscle tone and enhancement of lower extremity function. The presence of variability among individuals with SCI presents a distinct and advantageous prospect for AI to facilitate desired results and evaluate risk within this specific group of patients.

## Figures and Tables

**Table 1:** RAGT concise overview of the chosen papers’ key characteristics and bias risk.

Author, Year	Country	Total Sample (TS)	Study Design	Intervention	Outcome	Level of Injury	NOS
Hornby 2005 [22]	USA	30	RCT	The utilization of robotic assistance in BWSTT and therapist-assisted BWSTT. The intervention involved engaging in overground ambulation via a mobile suspension device for three 30 min weekly sessions over 8 weeks.	LEMS, 6MWT	AIS B, C, and D. The level of damage is located above the tenth thoracic vertebra (T10).	-
Wirz 2005 [23]	USA	20	Single group	The Lokomat (DGO) intervention consisted of an 8 week duration, with participants attending three to five sessions per week, each lasting 45 min.	AS, LEMS, 6MWT, 10MWT, and TUG	AIS C and D. The level of injury is at L1 or above.	7
Field 2011 [24]	USA	64	RCT	The participants engaged in a training regimen for 12 weeks, with a frequency of 5 days per week. The training program encompassed four distinct modalities, namely treadmill-based training with manual help (TM), treadmill-based training with stimulation (TS), overground training with motivation (OG), and treadmill-based training with robotic assistance (LR).	LEMS	AIS C and D. The level of damage is located at or above the tenth thoracic vertebra (T10).	-
Alcobendas 2012 [25]	Spain	75	RCT	The study consisted of a total of 40 sessions conducted over 8 weeks. Each session lasted for approximately 1 h and involved a Lokomat group intervention. Specifically, participants spent 30 min utilizing the Lokomat device within each session, followed by an additional 30 min of normal physical treatment. The overground group implemented a standardized biological treatment protocol for one hour.	VAS, LEMS, 6MWT, and 10MWT	AIS C and D. The range of injuries observed in the individual spans from the second cervical vertebra (C2) to the twelfth thoracic vertebra (T12).	-
Aach 2014 [26]	Germany	8	Pre-post experimental design	HAL had been used for 90 days, with a frequency of five weekly sessions.	LEMS, 6MWT, 10MWT, and TUG	ASIA A. Degree of damage: T8 to L2	7
del-Ama 2014 [27]	Switzerland	3	Pilot study	The Kinesis system was implemented during the first week, whereas no intervention was delivered the following week.	AS, VAS, 6MWT, and 10MWT	AIS A and D. Injuries impact the spinal levels encompassing L1 and L2.	8
Labruyère 2014 [28]	Switzerland	9	RCT	The first group underwent 16 sessions of RAGT using the Lokomat device, followed by an additional 16 strength training sessions. Group 2 received the intervention in reverse order.	VAS, LEMS, and 10MWT	AIS C and D. The extent of the injury ranges from the fourth cervical vertebra (C4) to the eleventh thoracic vertebra (T11).	-
Niu 2014 [29]	USA	40	RCT	The experimental group underwent twelve 1 h Lokomat training sessions over one month, whereas the control group did not receive any interventions.	TUG	AIS B, C, and D. The level of damage is located above the tenth thoracic vertebra (T10).	-
Shin 2014 [30]	South Korea	53	RCT	In four weeks, the RAGT group received three 40 min sessions per week of RAGT in addition to regular physiotherapy. The conventional group received physiotherapy twice daily, five days per week.	LEMS	AIS D. The level of injury is classified as upper motor neuron (UMN) involvement.	-
Varoqui 2014 [31]	USA	30	RCT	The Lokomat group participated in three weekly sessions for four weeks, each lasting one hour. The control group, on the other hand, did not receive any intervention.	6MWT, 10MWT, TUG	AIS C and D. The level of damage is located above the tenth thoracic vertebra (T10).	-
Duffell 2015 [32]	USA	56	RCT	The study involved allocating participants with an incomplete SCI into three groups: a control group receiving no intervention, a group receiving Lokomat intervention, and a group receiving tizanidine intervention.	TUG	AIS C and D The level of damage is located above the tenth thoracic vertebra (T10).	-
Lam 2015 [33]	Canada	15	RCT	The Lokomat-assisted BWSTT intervention was conducted for 45 min, three times a week, for three months.	6MWT, 10MWT	AIS C and D. Exclusion criteria encompassed individuals with lower motoneuron damage or lesion levels than T11.	-
Stampacchia 2016 [34]	Italy	21	Single group	The robotic exoskeleton (Ekso GT) exercise lasted approximately 40 min.	MAS, VAS	AIS A, B, and D. The observed lesions were located at the low cervical level (C7), dorsal level, and high lumbar level (L1–L2).	7
Mazzoleni 2017 [35]	Italy	7	Single group	The study consisted of 20 sessions, with a frequency of three sessions per week. The first set of sessions utilized a FES cycling system called Pegaso. It was followed by another group of 20 sessions, again with a frequency of three sessions per week, where participants used an overground robotic exoskeleton called the Ekso GT.	MAS, VAS, 6MWT, 10MWT, and TUG	AIS A. Injury severity: T4–T12	7
Watanabe 2019 [36]	Japan	2	Case report	HAL has been used 3–4 times weekly for eight sessions. It is performed with regular physical therapy, each lasting approximately 20–30 min.	MAS, LEMS	AIS C and D Injury severity: T8–T10, L1	7
Wirz 2017 [37]	USA	21	RCT	The intervention group received a training duration of 50 min per session, while the control group had a training duration of 25 min per session using the Lokomat device. Both groups underwent training sessions 3–5 days per week for a total period of 8 weeks.	10MWT	AIS B and C. C4 to T12 are affected.	-

AS: Ashworth Scale; MAS: Modified Ashworth Scale; VAS: Visual Analog Scale; 6MWT: 6 Minute Walk Test; 10MWT: 10 Meter Walking Test; LEMS: Lower Extremity Motor Score; TUG: Timed Up And Go Test; BWSTT: Body Weight-Supported Treadmill Training; HAL: Hybrid Assistive Limb; FES: Functional Electrical Stimulation; AIS: ASIA Impairment Scale; SCI: Spinal Cord Injury; NOS: Newcastle Ottawa Scale.

**Table 2:** ML concise overview of the chosen papers’ key characteristics and bias risk.

Author, Year	Country	Total Sample (TS)	Study Design	Intervention	Outcome	AIS Grade	NOS
DeVries 2019 [38]	Canada	862	Retrospective	The comparison of unsupervised MLA and LR, utilizing comprehensive neurological data for total admission, did not reveal any clinically significant disparities in functional prediction compared to previous models.	The F1-score has been demonstrated to possess greater reliability in evaluating algorithms than the area under the operating curve.	AIS A, B, C, and D	8
Torres 2021 [39]	USA	118	Retrospective	A similar network has been developed among patients to predict neurological recovery following spinal cord damage, focusing on MAP recorded before surgery.	The findings from the network analysis indicate that deviations from the optimal MAP range, either in the form of hypotension or hypertension, during surgical procedures are correlated with a reduced probability of achieving neurological recovery.	AIS A, B, C, D, and E	8
Agarwal 2022 [40]	USA	74	Retrospective	This study uses a deep-tree-based machine learning approach to evaluate the impact of intraoperative MAP and vasopressor administration on enhancing neurological outcomes in individuals with acute spinal cord injury.	An association between a MAP ranging from 80 to 96 mmHg and enhanced neurological function has been observed. Conversely, 93 min or more spent outside the MAP range of 76 to 104 mmHg had been associated with a worse outcome.	AIS A, B, C, D, and E	7

MLA: Machine Learning Algorithms; LR: Logistic Regression; MAP: Mean Arterial Pressure; NOS: Newcastle Ottawa Scale.

## Data Availability

This study did not include generating or analyzing new data. Sharing data is neither relevant nor appropriate to this article’s subject matter.
